# Myelin Oligodendrocyte Glycoprotein Antibody-Associated Disease (MOGAD): A Review of Clinical and MRI Features, Diagnosis, and Management

**DOI:** 10.3389/fneur.2022.885218

**Published:** 2022-06-17

**Authors:** Elia Sechi, Laura Cacciaguerra, John J. Chen, Sara Mariotto, Giulia Fadda, Alessandro Dinoto, A. Sebastian Lopez-Chiriboga, Sean J. Pittock, Eoin P. Flanagan

**Affiliations:** ^1^Neurology Unit, Department of Medical, Surgical and Experimental Sciences, University of Sassari, Sassari, Italy; ^2^Neuroimaging Research Unit, Division of Neuroscience, IRCCS San Raffaele Scientific Institute and Vita-Salute San Raffaele University, Milan, Italy; ^3^Department of Neurology and Center for Multiple Sclerosis and Autoimmune Neurology Mayo Clinic, Rochester, MN, United States; ^4^Department of Ophthalmology, Mayo Clinic, Rochester, MN, United States; ^5^Neurology Unit, Department of Neurosciences, Biomedicine, and Movement Sciences, University of Verona, Verona, Italy; ^6^Department of Neurology and Neurosurgery, McGill University, Montreal, QC, Canada; ^7^Department of Neurology, Mayo Clinic, Jacksonville, FL, United States; ^8^Department of Laboratory Medicine and Pathology, Mayo Clinic, Rochester, MN, United States

**Keywords:** MOG, NMOSD, neuromyelitis optica, multiple sclerosis, demyelinating diseases, false positive, differential diagnosis

## Abstract

Myelin oligodendrocyte glycoprotein (MOG) antibody-associated disease (MOGAD) is the most recently defined inflammatory demyelinating disease of the central nervous system (CNS). Over the last decade, several studies have helped delineate the characteristic clinical-MRI phenotypes of the disease, allowing distinction from aquaporin-4 (AQP4)-IgG-positive neuromyelitis optica spectrum disorder (AQP4-IgG+NMOSD) and multiple sclerosis (MS). The clinical manifestations of MOGAD are heterogeneous, ranging from isolated optic neuritis or myelitis to multifocal CNS demyelination often in the form of acute disseminated encephalomyelitis (ADEM), or cortical encephalitis. A relapsing course is observed in approximately 50% of patients. Characteristic MRI features have been described that increase the diagnostic suspicion (e.g., perineural optic nerve enhancement, spinal cord H-sign, T2-lesion resolution over time) and help discriminate from MS and AQP4+NMOSD, despite some overlap. The detection of MOG-IgG in the serum (and sometimes CSF) confirms the diagnosis in patients with compatible clinical-MRI phenotypes, but false positive results are occasionally encountered, especially with indiscriminate testing of large unselected populations. The type of cell-based assay used to evaluate for MOG-IgG (fixed vs. live) and antibody end-titer (low vs. high) can influence the likelihood of MOGAD diagnosis. International consensus diagnostic criteria for MOGAD are currently being compiled and will assist in clinical diagnosis and be useful for enrolment in clinical trials. Although randomized controlled trials are lacking, MOGAD acute attacks appear to be very responsive to high dose steroids and plasma exchange may be considered in refractory cases. Attack-prevention treatments also lack class-I data and empiric maintenance treatment is generally reserved for relapsing cases or patients with severe residual disability after the presenting attack. A variety of empiric steroid-sparing immunosuppressants can be considered and may be efficacious based on retrospective or prospective observational studies but prospective randomized placebo-controlled trials are needed to better guide treatment. In summary, this article will review our rapidly evolving understanding of MOGAD diagnosis and management.

## Introduction

During the last 15 years, the global concept of inflammatory demyelinating disorders of the central nervous system (CNS) has radically changed with the identification of specific autoantibody-associated conditions distinct from multiple sclerosis (MS), namely aquaporin-4 (AQP4)-IgG-positive neuromyelitis optica spectrum disorder (AQP4-IgG+NMOSD) and myelin oligodendrocyte glycoprotein (MOG)-IgG-associated disease (MOGAD) (1–4). Consensual refinements in the clinical-MRI characterization of these three disease entities has increased diagnostic precision, and allowed identification of important differences in pathophysiology, treatment response, and outcomes. Awareness of the specific features that define each demyelinating disorder is crucial for a correct diagnosis and timely initiation of an appropriate treatment (4).

In this review article we will summarize our current understanding of MOGAD, the most recently characterized demyelinating CNS disorder, and provide guidance for diagnosis and management. Although not representing the focus of this work, AQP4-IgG+NMOSD and MS will be discussed as comparison groups to highlight differences and similarities with MOGAD.

### History and Definitions: The Concept of NMOSD and MOGAD

There is often confusion among clinicians since both the terms NMOSD and MOGAD have been used in the literature to refer to the CNS demyelinating disorder associated with MOG-IgG. The term neuromyelitis optica (NMO) was first used in 1894 by Eugene Devic and his student Fernand Gault to describe a syndrome characterized by the simultaneous occurrence of bilateral optic neuritis (ON) and acute myelitis. Devic and Gault reviewed the literature at the time for similar cases and proposed the disease as a distinct entity, although the syndrome was regarded for decades by many as a more severe variant of MS, sometimes with different names worldwide (e.g., optic-spinal MS in Asia) (5). In 2004, Vanda Lennon and Brian Weinshenker published on a novel autoantibody that they identified in a cohort of patients with NMO but not in patients with MS, which they initially named NMO-IgG (1). The antibody was later found to target AQP4, the main water-channel protein in the CNS mostly expressed on astrocytic end-feet, and the name was changed to AQP4-IgG (6). It soon became clear that the spectrum of clinical-MRI manifestations related to AQP4-IgG extended beyond the exclusive involvement of optic nerves and spinal cord. Brain involvement was in fact recognized in a significant proportion of patients (7, 8), with area postrema syndrome (i.e., intractable nausea, vomiting, and hiccups) being one of the cardinal manifestations of the disease (9). Patients with AQP4-IgG could also present with partial forms of isolated myelitis or optic neuritis that did not meet the former NMO criteria. As a consequence, the definition of NMO spectrum disorders (NMOSD) was created to include the different clinical-MRI syndromes associated with the novel antibody (10). In 2015, NMOSD diagnostic criteria were published by a panel of experts (11). In addition to the main diagnosis based on AQP4-IgG detection, these criteria introduced the concept of seronegative NMOSD defined by more stringent clinical-MRI requirements based on the core clinical-MRI manifestations observed in patients with AQP4-IgG. However, the actual identification of patients meeting these criteria despite testing negative for AQP4-IgG raised the possibility that other autoantibodies could account for similar syndromes, which was later found to be true for MOG-IgG.

The historical scientific interest for the MOG protein dates back to the 1980's when it was first identified as a major potential autoantibody target of CNS myelin in models of experimental autoimmune encephalomyelitis (12). In 2003, MOG-IgM detected by Western Blot was proposed as a biomarker to predict conversion from clinically isolated syndrome to definite MS (13). However, a number of subsequent studies conducted with similar testing assays using denaturated MOG proteins (e.g., ELISA) showed that MOG-IgG was not disease-specific, being detectable with similar frequency in patients with MS, other demyelinating CNS disorders, and unaffected controls (14, 15). In 2007, a seminal study by O'Connor et al. showed that laboratory assays expressing MOG in its tridimensional conformational form (rather than linear epitopes targeted on denaturated proteins as with other assays) identified a sub-set of conformation-sensitive MOG-IgG in patients with acute disseminated encephalomyelitis (ADEM) or optic neuritis, but not in patients with MS (2). Subsequent studies using cell-based assays expressing human full length MOG on mammalian cells confirmed the detection of MOG-IgG in patients with non-MS demyelinating CNS disorders (16, 17), including 30–70% of patients with seronegative NMOSD (18–21). The spectrum of clinical-MRI manifestations associated with MOG-IgG is now recognized to extend beyond the NMOSD phenotype and the disease is referred to as MOGAD (22–24).

To avoid confusion and overlaps between clinical-MRI phenotypes (e.g., NMOSD, ADEM), an antibody-based nomenclature is preferred. As an example, the disease associated with MOG-IgG (i.e., MOGAD) can manifest with different clinical-MRI phenotypes, such as optic neuritis, myelitis, encephalitis, ADEM, NMOSD, or combinations thereof. On the contrary, an NMOSD phenotype can be seen in patients with AQP4-IgG, MOG-IgG, or rare double seronegative cases. In this review article, we will refer to the spectrum of clinical-MRI phenotypes associated with AQP4-IgG and MOG-IgG as AQP4-IgG+NMOSD and MOGAD, respectively.

### Pathology and Pathophysiology

The MOG protein is selectively expressed in the CNS where it represents approximately 0.05% of the total myelin proteins. Its location on the outermost myelin sheath layers and oligodendrocyte cell surface makes it directly accessible to MOG-IgG, although the exact role of MOG-IgG in the pathophysiology of MOGAD remains unclear (3, 25, 26).

The neuropathological hallmarks of MOGAD include perivenous and confluent white matter demyelination, MOG-dominant myelin loss, intracortical demyelination, predominant CD4+ T-cell and granulocytic inflammation, complement deposition within active white matter lesions, partial axonal preservation, and reactive gliosis (27–29). Most of these features resemble the pathology of ADEM, which is characterized by perivenous and confluent diffuse inflammation and demyelination, and is now known to be associated with MOG-IgG in about 50% of cases (30).

These aspects are also partially overlapping with the neuropathological characteristics of MS, and in particular are reminiscent of “MS pattern II,” with IgG and activated complement deposits in active demyelinating lesions (28, 31–33). However, the predominant intracortical location of cortical demyelinating lesions, together with predominant CD4+ rather than CD8+ T-cells/B cells inflammatory infiltrates help distinguish MOGAD from MS. In addition, slowly expanding demyelinated plaques, which are a common feature in MS, are usually absent in MOGAD. In MOGAD pathology, AQP4 is well-preserved and associated with hypertrophic reactive astrocytes with no evidence of complement deposition in sites of AQP4 expression and relative preservation of oligodendrocyte, which are aspects that clearly distinguishes MOGAD and AQP4-IgG+NMOSD (27, 29, 34, 35). These pathologic features of MOGAD are also observed in fulminant cases, where, in addition, superimposed ischemic damage, necrosis, astrocyte and axonal damage can be observed (36).

Of note, the same neuropathological features observed in MOGAD seropositive cases are detected in rare seronegative patients with exclusive intrathecal MOG-Abs synthesis (CSF MOG-IgG positive patients who are seronegative for MOG-IgG), where confluent demyelination with perivascular accentuation, gliosis, relative axonal sparing, and complement deposition are observed. These cases also show an almost equal presence of T- and B-cells, possibly representing a specific feature of CSF MOG-Abs synthesis (37, 38). Altogether, these neuropathological characteristics suggest that humoral mechanisms are involved in the pathogenesis of the disease, through processes of antibody/complement mediated demyelination. After binding of the antibodies to the surface of myelin, the damage might be induced by complement (activated by the IgG1 subclass of MOG-Abs) or antibody-dependent cellular cytotoxicity supported by activated innate effector cells, but the specific pathogenesis is still being elucidated (33, 39, 40).

### Epidemiology

There are limited population-based data on the epidemiology of MOGAD in relation to different geographical areas and ethnicities. Two major limitations for a robust assessment of MOG-IgG seroprevalence in a population include: (1) MOG-IgG titer may drop to undetectable after disease attacks (i.e., MOGAD patients tested during remission may result negative and erroneously be excluded from a study); and (2) screening of large populations for epidemiology purposes increases the risk of false positive results (see also “*Atypical clinical-MRI phenotypes and risk of false positivity*” below). Similar problems are not encountered with AQP4-IgG+NMOSD for which antibody positivity persists over time and the risk of false positive results using cell-based assay technique is negligible (19).

MOGAD has a female-to-male ratio of approximately 1:1, which is different from the female predominance typically observed in other autoimmune/immune-mediated disorders including AQP4-IgG+NMOSD (9:1) (41), and MS (3:1) (42). The relative frequency of MOGAD among demyelinating CNS disorders seems higher in children (<18 years of age) than in adults, although any age can potentially be affected. A study by de Mol et al. found a 7% frequency of MOG-IgG positivity among 1,414 samples (1,277 patients) sent to the central reference laboratory in the Netherlands between February 2014 and December 2017 for routine diagnostics. In the same study, the estimated annual incidence was 1.6/million person-years (children, 3.1/million; adults, 1.3/million). Overall, this is similar to that of AQP4-IgG+NMOSD (0.4–7.3/million) (41, 43), and markedly lower than that of MS (7–144/million) (42, 44), accepting a wide geographical variability. While no clear ethnic preponderance has been demonstrated for MOGAD, its frequency in Caucasian populations seems two to three-fold higher compared to that of AQP4-IgG+NMOSD (45, 46), while the latter might be more represented than MOGAD among African-Americans or Afro-caribbeans (41, 43, 47). In Asia, AQP4-IgG+NMOSD is more prevalent compared to the Caucasian populations although the frequency of MOGAD might be even higher. One study assessing the frequency of autoantibodies among 726 serum samples consecutively referred to the National Hospital of Sri Lanka for suspected demyelinating CNS disorder, found the frequency of MOG-IgG (17%) was 3.5 times higher than that of AQP4-IgG (5%) (46). The increased relative frequency of antibody-mediated demyelinating CNS disorders in Asia is counterbalanced by a lower MS prevalence compared to Caucasians (48, 49).

**Figure 1:**
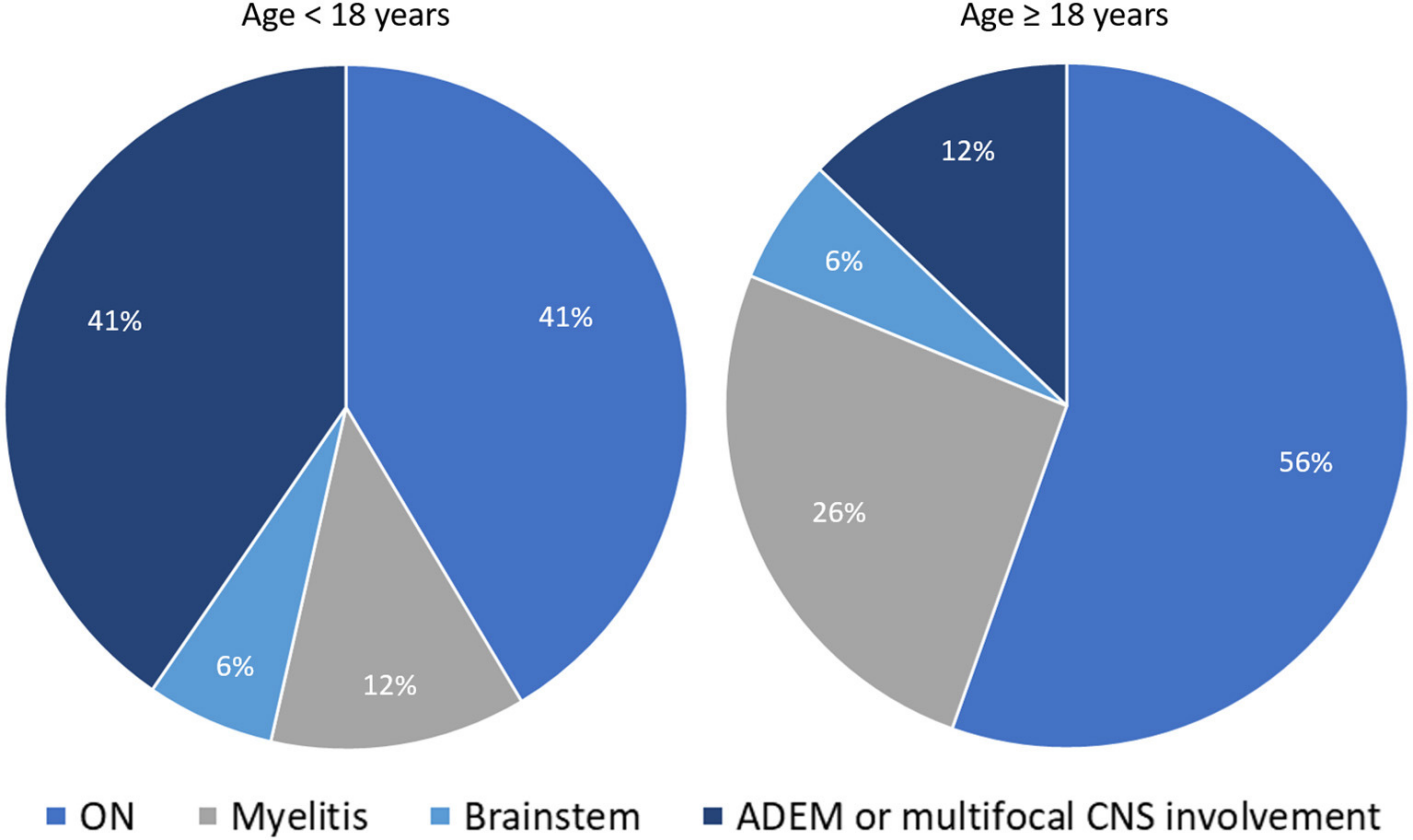
Distribution of different disease phenotypes at MOGAD presentation stratified by age. The two pie-charts show the relative frequency of different disease phenotypes at MOGAD onset in patients below 18 years of age and older: ON (optic neuritis, unilateral or bilateral); myelitis; brainstem syndromes; ADEM (acute disseminated encephalomyelitis), or multifocal central nervous system involvement without encephalopathy. Note that the frequency of ADEM is significantly higher in younger patients. The reported frequencies are abstracted from Cobo-Calvo et al. (52).

The expected frequency of MOG-IgG positivity in clinical practice also varies based on the presenting clinical phenotype. In a population based-study of patients with new-onset ON conducted in Olmsted County (Minnesota, United States), the frequency of MOGAD and AQP4-IgG+NMOSD ON were 5 and 3%, respectively; with MS (57%) and idiopathic ON (29%) being the most common final diagnoses (50). Another population-based study in Olmsted County on patients with isolated idiopathic transverse myelitis (based on 2002 ITM diagnostic criteria) found AQP4-IgG-related myelitis to be twice as common as MOG-IgG-related myelitis, although both antibody-positive etiologies accounted for only 14% of total incident cases (51). This frequency increased to 50% when only cases with longitudinally extensive transverse myelitis were considered (51). Lastly, in patients with ADEM the overall frequency of MOG-IgG positivity far exceeds that of AQP4-IgG positivity, observed in approximately 50% and 5% of cases, respectively, with an important age-related variability of disease phenotypes (23, 30). In particular, ADEM or multifocal CNS involvement are four times less common in adults compared to pediatric MOGAD patients (in whom they represent the most common disease phenotype along with ON). Figure 1 shows the relative frequency of different disease phenotypes at MOGAD presentation stratified by age (52).

## Clinical-MRI Attributes

**Figure 2:**
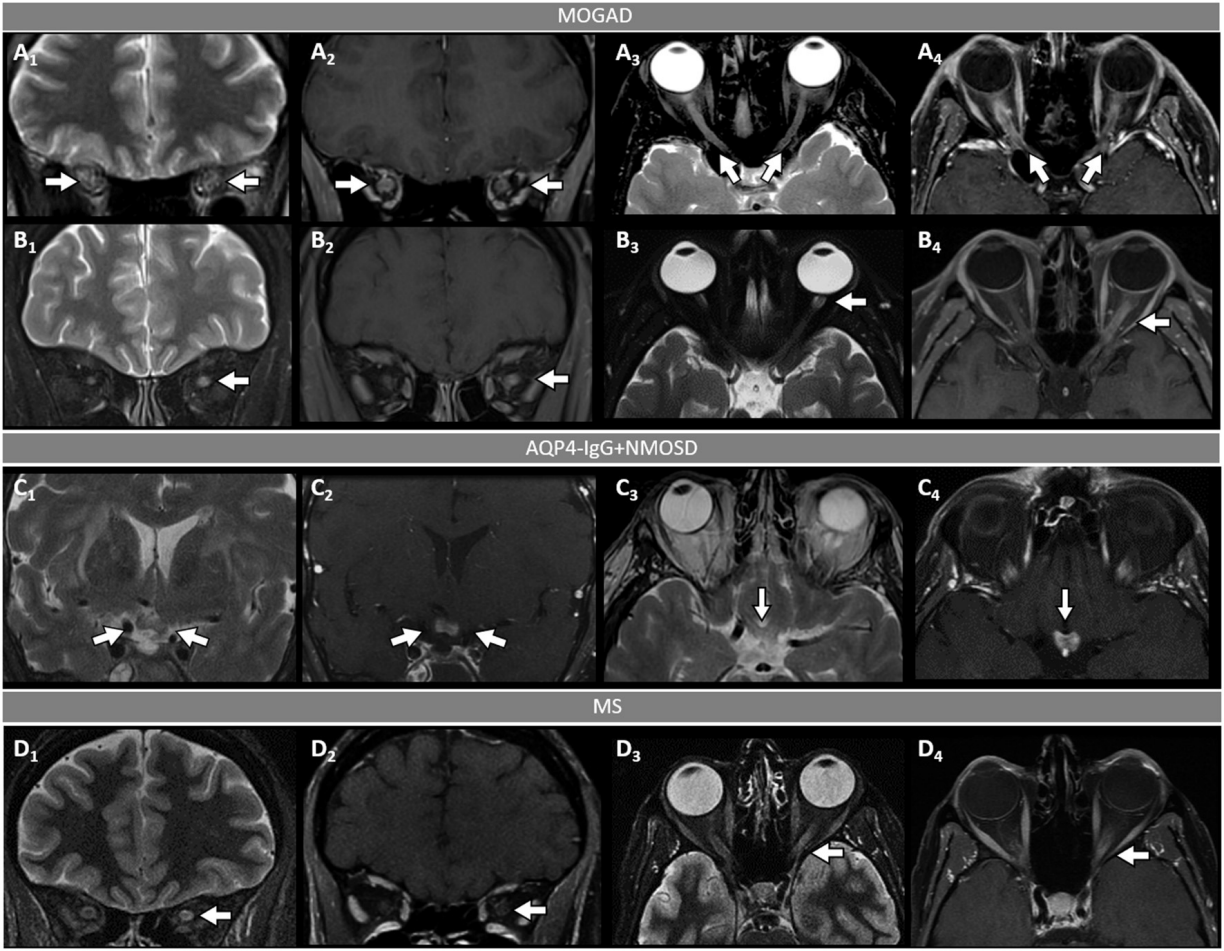
Orbital fat-saturated MRI in optic neuritis with MOGAD, AQP4-IgG+NMOSD, and MS. [**(A,B)**: MOGAD] Bilateral optic nerve sheath thickening (A_1_, coronal view, arrows) and optic nerve T2-hyperintensity (A_3_, axial view, arrows) on T2-weighted images, and corresponding longitudinally extensive optic nerve and sheath enhancement on T1-post gadolinium images (A_2_, coronal view, arrows, and A_4_, axial view, arrows). Unilateral left optic nerve T2-hyperintensity on T2-weighted images (B_1_, coronal view, arrow, and B_3_, axial view, arrow) and longitudinally extensive enhancement of the left optic nerve on T1-post-gadolinium images (B_2_, coronal view, arrow, and B_4_, axial view, arrow). [**(C)**: AQP4-IgG+NMOSD] Bilateral optic chiasm T2-hyperintensity on T2-weighted images (C_1_, coronal view, arrows, and C_3_, axial view, arrow) and optic chiasm enhancement on T1-post-gadolinium images (C_2_ coronal views, arrow, and C_4_, axial view, arrow). [**(D)**: MS] Unilateral left optic nerve T2-hyperintensity on T2-weighted images (D_1_, coronal view, arrow, and D_3_, axial view, arrow), with a corresponding short segment of gadolinium-enhancement on T1-post-gadolinium images (D_2_, coronal view, arrow, and D_4_, axial view, arrow). MOGAD, myelin oligodendrocyte glycoprotein antibody-associated disease; AQP4-IgG+NMOSD, aquaporin-4-IgG seropositive neuromyelitis optica spectrum disorder; MS, multiple sclerosis. Definitions: Longitudinally extensive gadolinium enhancement= enhancement involving >50% the length of the optic nerve. Short-segment gadolinium enhancement= enhancement involving <50% the length of the optic nerve.

**Figure 3:**
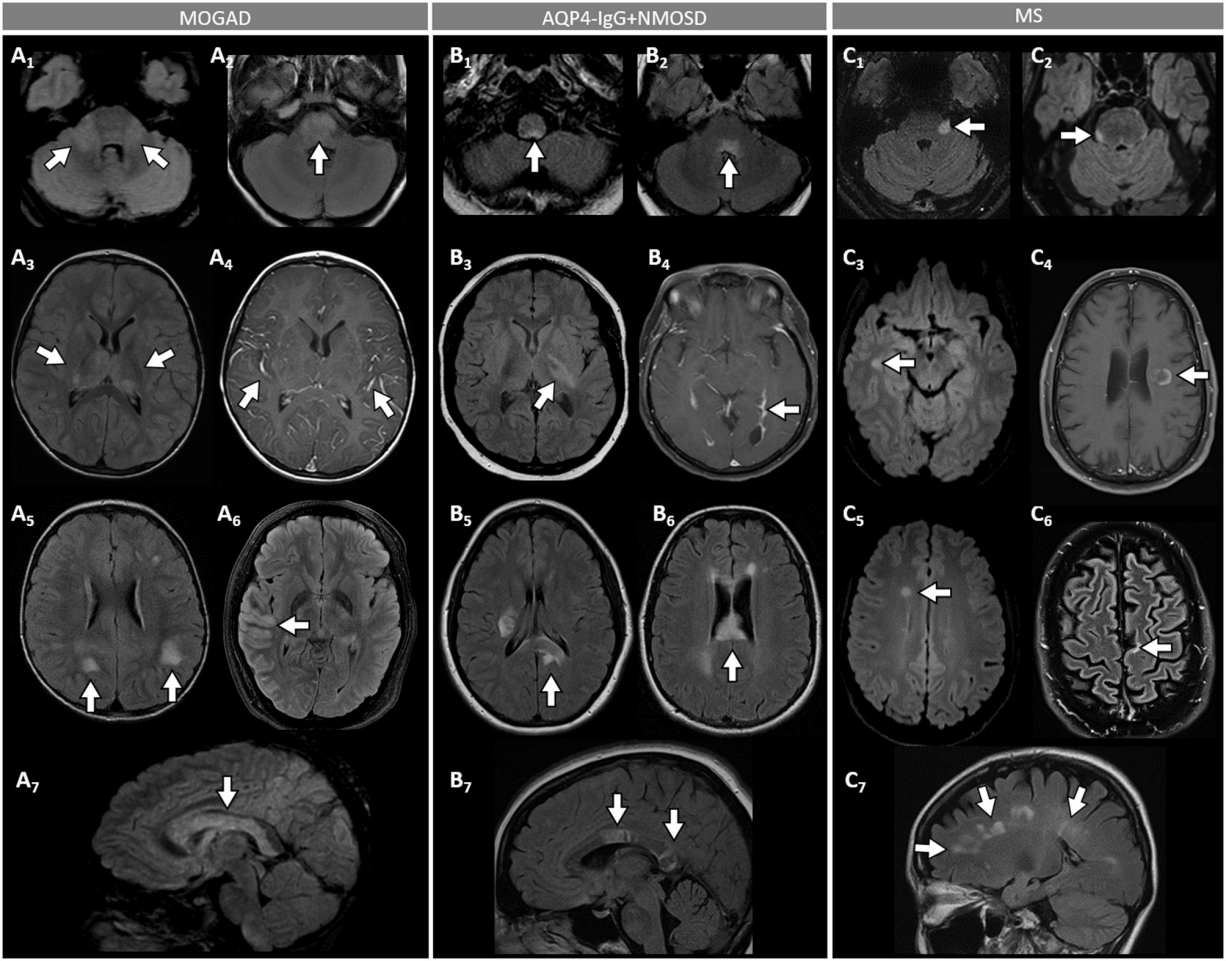
Brain MRI features in patients with MOGAD, AQP4-IgG+NMOSD, and MS. [**(A)**: MOGAD] T2-hyperintense lesions on axial FLAIR images diffusely involving the middle cerebellar peduncles bilaterally (A_1_, arrows), pons (A_2_, arrow), and bilateral thalami (A_3_, arrows); while extensive leptomeningeal enhancement is noted on axial T1-post gadolinium images (A_4_, arrows); axial FLAIR images reveal T2-hyperintensities that are poorly marginated or “fluffy” in the hemispheric white matter (A_5_, arrows), with thickening of the right temporal-parietal cortex (A_6_, arrow), and diffuse involvement of the corpus callosum on sagittal view (A_7_, arrow). [**(B)**: AQP4-IgG+NMOSD] Axial FLAIR images show T2-hyperintense lesions in the area postrema (B_1_, arrow), dorsal pons adjacent to the 4th ventricle (B_2_, arrow), and left corticospinal tract at level of the internal capsule (B_3_, arrow); axial T1-post gadolinium image reveals linear ependymal enhancement in the posterior horn of the left lateral ventricle (B_4_, arrow); FLAIR images reveal T2-hyperintense lesions involving the splenium of the corpus callosum (B_5_, arrow, axial view) and a diffuse “marble pattern” hyperintensity of the corpus callosum (B_6_, axial view, arrow, and B_7_, sagittal view, arrows). [**(C)**: MS] Axial FLAIR images reveal small foci of T2-hyperintensity involving the pons at the emergence of the left trigeminal nerve (C_1_, arrow), and on its ventral-right surface (C_2_, arrow); axial FLAIR images reveal periventricular T2-hyperintense lesions in the inferior temporal pole (C3, arrow) and frontal horn with an ovoid appearance (C5, arrow); on axial T1-post gadolinium images an incomplete ring enhancing white matter lesion is shown (C_4_, arrow); FLAIR images reveal a juxtacortical T2-hyperintense lesion (C_6_, axial view, arrow) and Dawson's fingers T2-hyperintense lesions (C_7_, sagittal view, arrows). MOGAD, myelin oligodendrocyte glycoprotein antibody-associated disease; AQP4-IgG+NMOSD, aquaporin-4-IgG seropositive neuromyelitis optica spectrum disorder; MS, multiple sclerosis; FLAIR, fluid attenuated inversion recovery.

**Figure 4:**
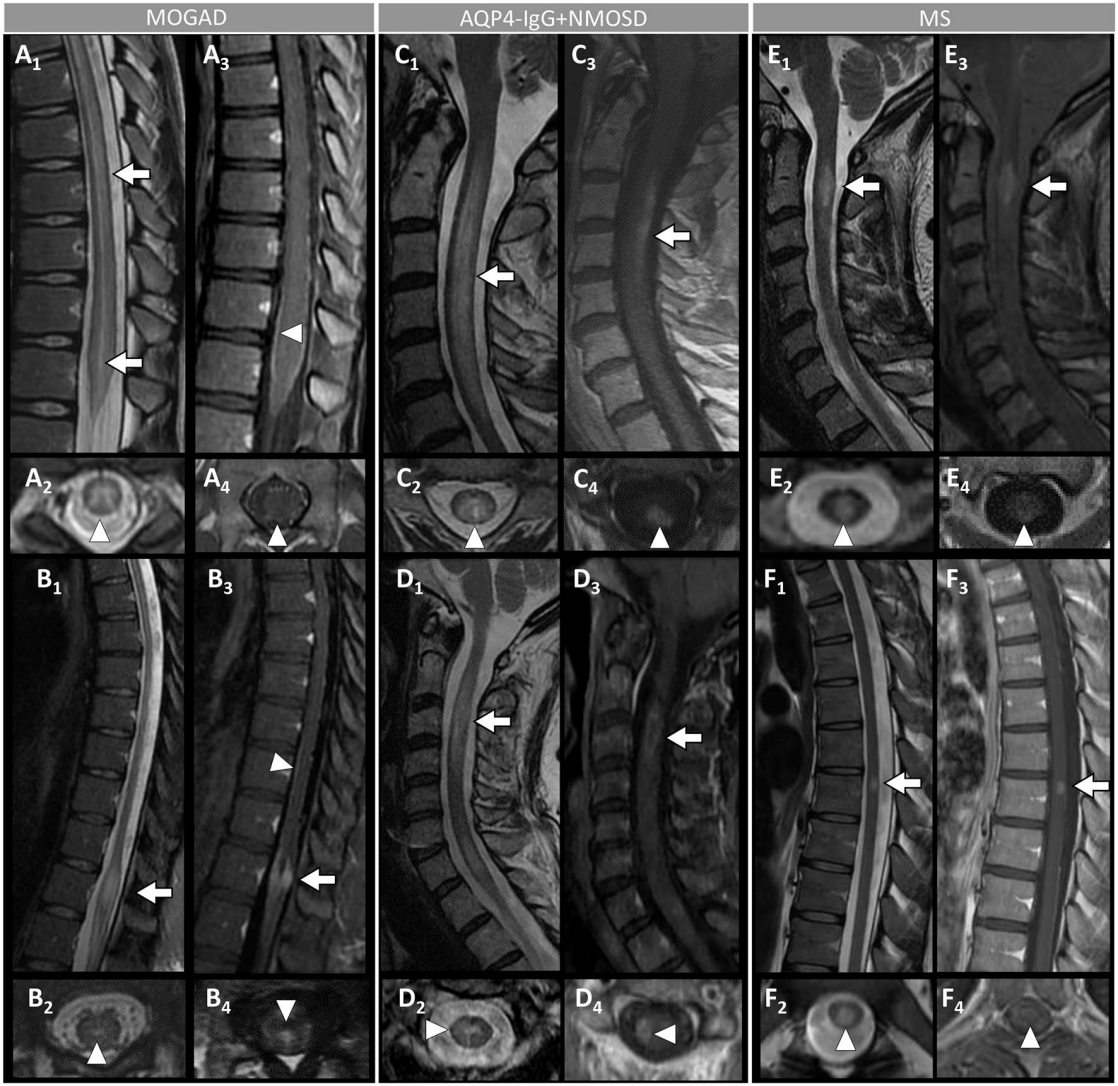
Spinal cord MRI features in patients with MOGAD, AQP4-IgG+NMOSD, and MS. [**(A,B)**: MOGAD] A longitudinally-extensive T2-hyperintense lesion involving the conus medullaris on sagittal images (A_1_, arrows), with T2-hyperintensity restricted to the gray matter forming a H-sign on the axial view (A_2_), accompanied by leptomeningeal enhancement but no spinal cord parenchymal enhancement on T1-post-gadolinium images (A_3_, sagittal view, arrowhead, and A_4_, axial view, arrowhead); a sagittal short T2-hyperintense lesion involving the conus (B_1_, arrow), centrally located on axial images (B_2_, arrowhead), with corresponding gadolinium-enhancement (B_3_, arrow) and leptomeningeal enhancement on T1-post-gadolinium images (B_3_, sagittal view, arrowhead, and B_4_, axial view, arrowhead). [**(C,D)**: AQP4-IgG+NMOSD] A sagittal longitudinally-extensive T2-hyperintense cervical cord lesion with spinal cord swelling (C_1_, arrow), gray matter involvement and T2-“bright spotty sign” on axial view (C_2_, arrowhead), and gadolinium-enhancement on T1-post-gadolinium images (C_3_, sagittal view, arrow, and C_4_, axial view, arrowhead); a sagittal longitudinally extensive T2-hyperintense cervical cord lesion (D_1_, arrow), predominantly involving the right hemi-cord on axial view (D_2_, arrowhead), accompanied by ring enhancement (D_3_, arrow; D_4_, arrowhead) on T1-post-gadolinium images. [**(E,F)**: MS] A sagittal short T2-hyperintense cervical cord lesion (E_1_, arrow) involving central spinal cord (E_2_, arrowhead), showing homogeneous gadolinium-enhancement on T1-post-gadolinium images (E_3_, sagittal view, arrow, and E_4_, axial view, arrowhead); a sagittal short T2-hyperintens thoracic spinal cord lesion (F_1_, arrow), involving the gray matter and the dorsal white matter tracts (F_2_, arrowhead), with homogeneous enhancement on T1-post-gadolinium images (F_3_, sagittal view, arrow, and F_4_, axial view, arrowhead). MOGAD, myelin oligodendrocyte glycoprotein antibody-associated disease; AQP4-IgG+NMOSD, aquaporin-4-IgG seropositive neuromyelitis optica spectrum disorder; MS, multiple sclerosis. Definitions: Longitudinally extensive spinal cord lesion= lesion involving ≥3 vertebral segments. Short spinal cord lesion= lesion involving <3 vertebral segments.

The spectrum of clinical-MRI manifestations of MOGAD is more heterogeneous when compared to other CNS demyelinating disorders, and important differences exist when the disease is studied during or after acute attacks (see also “*Attack-related manifestations*” and “*Post-attack MRI characteristics, disease course, and outcomes*” below). In general, MOGAD is characterized by severe clinical attacks accompanied by large or longitudinally extensive T2-lesions on brain, spine, or orbit MRI, sometimes described as “fluffy” due to the often poorly defined margins particularly compared to lesions in approximately MS that are well circumscribed (53, 54). Acute gadolinium enhancement of at least one lesion (generally considered an indirect hallmark of active inflammation on MRI) is seen in 50–75% of patients with brain and myelitis attacks, while with MS it is more consistently evident (55). In contrast, optic nerve enhancement is seen during almost all ON attacks and can characteristically involve the optic nerve sheath or peribulbar fat, which has been termed perineural enhancement (56, 57). Notably, the initial brain or spinal cord MRI in MOGAD can be normal in up to 10% of cases despite severe acute disability (58–60). In these patients, somatosensory evoked potentials or repeat MRI within days may help confirm involvement of the affected CNS region (59). Table 1 summarizes and compares the main clinical, laboratory, and MRI features generally observed in patients with MOGAD, AQP4-IgG+NMOSD, and MS; while examples of typical MRI abnormalities seen in MOGAD patients with ON, brain, and myelitis attacks compared to AQP4-IgG+NMOSD and MS are shown in Figures 2–4, respectively (61, 62).

**Table 1:** Comparison of demographics, clinical, laboratory and MRI characteristics in MOGAD compared to AQP4-IgG+NMOSD, and MS.

	**MOGAD**	**AQP4-IgG-NMOSD**	**MS**
**Demographics**			
Most commonly affected age	0–40	30–50	20–40
Sex (F:M)	1:1	9:1	2–3:1
Ethnicity	No clear racial predominance	Any, African-American and Asian at higher risk	Any, mostly Caucasian
**Clinical features**			
Antecedent infection/immunization	Common	Rare	Rare
Disease course	Relapsing (50%) or monophasic (50%); a progressive course is rare	Generally relapsing; a progressive course is extremely rare	Relapsing (85%) or progressive from onset (15%)
Optic neuritis	+++	+++	++
Myelitis	++	+++	+++
Area postrema syndrome	Rare	++	Rare
Encephalopathy	++	Rare	Rare
Seizures	+	Rare	Rare
**CSF**			
Oligoclonal bands	<20% (transient)	<20% (transient)	>85% (persistent)
White cell count >50/μl	35%	13–35%	Rare
**MRI**			
**Acute attacks**			
Optic nerve	Uni-/bilateral, long lesions (>50%), mainly anterior segments, perineural enhancement	Uni-/bilateral, mainly posterior segments including chiasm	Generally unilateral, short lesions, mainly along the intraorbital tract
Spinal cord	Longitudinally extensive (60%), generally >1 lesion, conus involved, H-sign axially, enhancement in 50%	Longitudinally extensive (85%), single lesion. Central/diffuse on axial images. Elongated ring or patchy enhancement	Multiple short lesions; periphery of cord. Ring or nodular enhancement
Brain	ADEM-like, “fluffy” lesions in both white and deep gray matter; extensive involvement of cerebellar peduncles. Cortical hyperintensity may occur	Often non-specific; area postrema, peri- 3^rd^/4^th^ ventricle, corticospinal tracts, sometimes extensive white matter lesions	Ovoid periventricular, infratentorial, juxtacortical. Central vein sign. Ring enhancement
Initially normal MRI	Up to 10%	Rare	Rare
**Post-attack MRI**			
T2-lesion resolution	50–80%	Rare	Rare
New asymptomatic T2-lesions occurrence	Rare	Rare	Common
Residual T1-hypointensity	Rare	Common	Common
Persistent acute gadolinium enhancement >6 months	Rare	Rare	Rare
**Treatment**			
Acute	IVMP; PLEX if severe episode; IVIg possible alternative, especially for children	IVMP; low threshold to follow with PLEX	IVMP; PLEX reserved for the rare very severe attacks
Recovery	Generally good despite severe attacks	Often incomplete	Generally good
Maintenance	No proven treatments with class 1 evidence. Common empiric options include mycophenolate, rituximab, periodic IVIg, tocilizumab, oral steroids	Class 1 evidence for: eculizumab, inebilizumab, rituximab and satralizumab; other: azathioprine, mycophenolate, oral steroids, tocilizumab	Large variety of MS medications proven to be effective in class 1 studies

*ADEM, acute disseminated encephalomyelitis; AQP4-IgG+NMOSD, aquaporin-4-IgG-positive neuromyelitis optica spectrum disorder; CSF, cerebrospinal fluid; IVIg, intravenous immunoglobulins; IVMP, intravenous methyl-prednisone; MOGAD, myelin oligodendrocyte glycoprotein antibody-associated disease MS, multiple sclerosis; PLEX, plasma exchange*.

### Attack-Related Manifestations

The different clinical-MRI phenotypes seen in patients with MOGAD attacks can occur in isolation or in various combinations, with variable frequency in children and adults (30, 52, 63). For example, ADEM is more common in children while optic neuritis is the most common manifestation of MOGAD in adults (Figure 1).

- Optic neuritis is the most common manifestation in MOGAD and frequently occurs in isolation. It is recurrent or bilateral/simultaneous in approximately 50% of cases (57), and sometimes is steroid-dependent with a chronic relapsing inflammatory optic neuropathy (CRION)-like phenotype (64). Patients typically present with pain with extraocular movements and/or optic disc oedema (85%) on fundoscopic examination that may be severe and sometimes accompanied by hemorrhages. The visual deficit at nadir is usually severe (count fingers in most cases), but fortunately recovery is usually good leading to significantly better visual outcomes than AQP4-IgG+NMOSD (57). Up to 50% of patients complain of a new-onset, often severe periorbital and fronto-temporal headache a few days before the visual deficit (65). On MRI, an extensive involvement of the optic nerve for most of its length on T2 or post-gadolinium T1-weighted images is characteristic (~85% of patients), with enhancement of the optic nerve sheath (perineural enhancement) often extending to the surrounding orbital tissue observed in 50% of cases (Figure 2) (57). Involvement of the optic chiasm, originally considered characteristic of AQP4-IgG+NMOSD, can be seen in a minority of MOGAD patients but rarely in isolation, and generally accompanied by extensive involvement of the optic nerves (66, 67). Bilateral longitudinally extensive enhancement of the optic nerve not involving the chiasm is suggestive of MOGAD (56).
- An ADEM or ADEM-like phenotype with multifocal CNS involvement are more common in children but can occur at any age (Figure 1) (52, 68). Patients may present with encephalopathy, focal deficits referable to the brain, or have asymptomatic brain abnormalities in the context of other MOGAD manifestations (e.g., ON or myelitis). The severity of episodes can be such that patients require ventilatory support with cerebral attacks in up to 3% (69). Brain MRI usually shows multiple large T2-abnormalities variably affecting the supratentorial white matter, the cortex, and/or the deep gray nuclei; unilateral or bilateral thalamic and basal ganglia signal abnormalities are common (Figure 3) (54). Confluence of large symmetrical lesions bilaterally may sometimes occur and mimic a leukodystrophy, especially in pediatric patients (70). On the contrary, solitary brain lesions are less common (54). The corticospinal tracts are often affected at the internal capsule or midbrain peduncle level, sometimes bilaterally mimicking Behcet's disease or genetic/metabolic disorders (71). The central vein sign, a highly specific finding in MS brain lesions (72), is uncommon in MOGAD and AQP4-IgG+NMOSD (73). When present, gadolinium enhancement of lesions is generally nonspecific (e.g., the ring or open-ring enhancement typically seen in MS lesions is uncommon in MOGAD) (62).
- Brainstem involvement rarely occurs in isolation and can be asymptomatic in up to 40% of cases (Figure 1) (74). Diffuse midbrain, pons or medulla T2-hyperintense lesions are seen in approximately 20% of cases, which are different from the short focal T2-lesions in MS. Unilateral or bilateral large poorly demarcated middle cerebellar peduncle lesions are often seen in MOGAD and help discriminate from AQP4-IgG+NMOSD or MS (Figure 3) (74). Rarely, patients may present clinically with intractable nausea, vomiting, or hiccups characteristic of the area postrema syndrome accompanied by T2-lesions in the region of the area postrema (a finding more typical of AQP4-IgG+NMOSD). More frequently, nausea and vomiting in MOGAD occur in the setting of ADEM and multifocal brain lesions, rather than in association with discrete area postrema lesions (75).
- Spinal cord involvement in MOGAD is frequently severe with paraparesis requiring a gait aid, and/or bladder dysfunction requiring catheterization at myelitis nadir. On MRI, spinal cord T2-lesions are typically longitudinally extensive spanning ≥3 contiguous vertebral body segments, although shorter lesions may coexist (as opposed to patients with AQP4-IgG+NMOSD, in whom a single longitudinally extensive lesion is noted in the majority of patients). Single or multiple short lesions can occur in MOGAD but are uncommon and should always raise the suspicion for a false MOG-IgG positivity in the context of MS, particularly when the lesions are peripherally located on axial spinal cord MRI (60, 76, 77). The conus medullaris is more frequently involved in MOGAD compared to AQP4-IgG+NMOSD (53). MOGAD myelitis T2-lesions often predominantly affect the ventral part of the spinal cord on sagittal images (“ventral sagittal line”), and/or the central gray matter on axial images (“H-sign”) in about a third (Figure 4) (53). Although highly suggestive of MOGAD, these signs are not 100% specific and can be seen in other myelopathies (*e.g*., acute flaccid myelitis, viral myelitis, idiopathic myelitis) (60, 78). Similar to brain lesions, acute gadolinium enhancement is often nonspecific, differing from other myelitis for which characteristic enhancement patterns have been described (e.g., “elongated ring” in AQP4-IgG+NMOSD, dorsal subpial enhancement and “trident sign” in spinal cord sarcoidosis) (78– 81). Leptomeningeal enhancement accompanying the myelitis can occur, and may be more common in children (60).
- Cerebral cortical encephalitis is a less common phenotype, also known as FLAMES (unilateral cortical FLAIR-hyperintense Lesions in Anti-MOG-associated Encephalitis with Seizures), characterized by encephalopathy, seizures, headache, marked CSF pleocytosis, and cortical hyperintensity on FLAIR images (82, 83). The seizures may evolve into status epilepticus and require ventilatory support (69). The cortical hyperintensity is generally unilateral but can be bilateral and accompanied by leptomeningeal enhancement in the affected brain region (Figure 3). The cortical hyperintensity can be the only MRI abnormality or occur in association with other brain and/or spinal cord lesions typical of MOGAD (84). Unilateral leptomeningeal enhancement has also been reported in patients with encephalitis and normal cortical signal on FLAIR images (85). The main differential of the FLAMES phenotype on MRI is with meningitis, subarachnoid hemorrhage, and CNS vasculitis (84, 86).
- Patients with MOGAD may rarely present with concomitant peripheral nervous system involvement and sometimes fulfill the criteria for combined central and peripheral demyelination (87). The peripheral neuropathy in these cases remains of unclear significance given the exclusive presence of MOG in the CNS in humans, although MOG mRNA transcripts have been detected in the peripheral nerves of rodents and primates (87, 88). Cranial nerve involvement beyond the optic nerve has also been reported (89).

### Post-attack MRI Characteristics, Disease Course, and Outcomes

**Figure 5:**
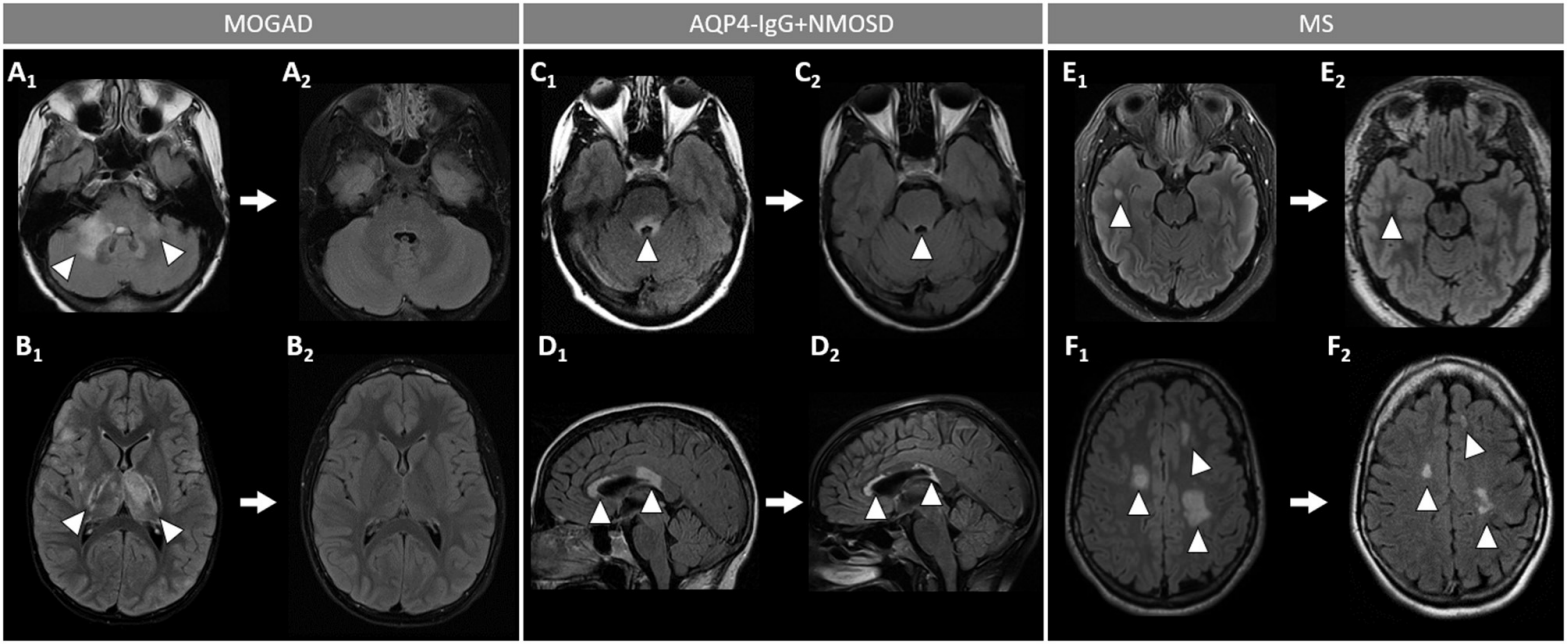
Brain MRI lesion evolution in patients with MOGAD, AQP4-IgG+NMOSD, and MS on FLAIR images. [**(A,B)**: MOGAD] Acute bilateral large T2-hyperintensities of middle cerebellar peduncles (right > left) (A_1_, arrowheads), and in the thalamus and cortico-spinal tract bilaterally (B_1_, arrowheads). All T2-lesions had resolved completely by the time of follow-up MRI (A_2_, B_2_). [**(C D)**: AQP4-IgG+NMOSD] Acute T2-hyperintense lesion around the 4th ventricle (C_1_, arrowhead), resolving to nearly undetectable at follow-up MRI (C_2_, arrowhead); and lesions involving the corpus callosum (D_1_, arrowheads), undergoing reduction in size without resolution on follow-up MRI (D_2_, arrowheads). [**(E,F)**: MS] Acute T2-hyperintense white matter lesions (E_1_, F_1_, arrowheads), reduced in size but still visible on follow-up MRI (E_2_, F_2_, arrowheads). MOGAD, myelin oligodendrocyte glycoprotein antibody-associated disease; AQP4-IgG+NMOSD, aquaporin-4-IgG seropositive neuromyelitis optica spectrum disorder; MS, multiple sclerosis. FLAIR, fluid attenuated inversion recovery.

**Figure 6:**
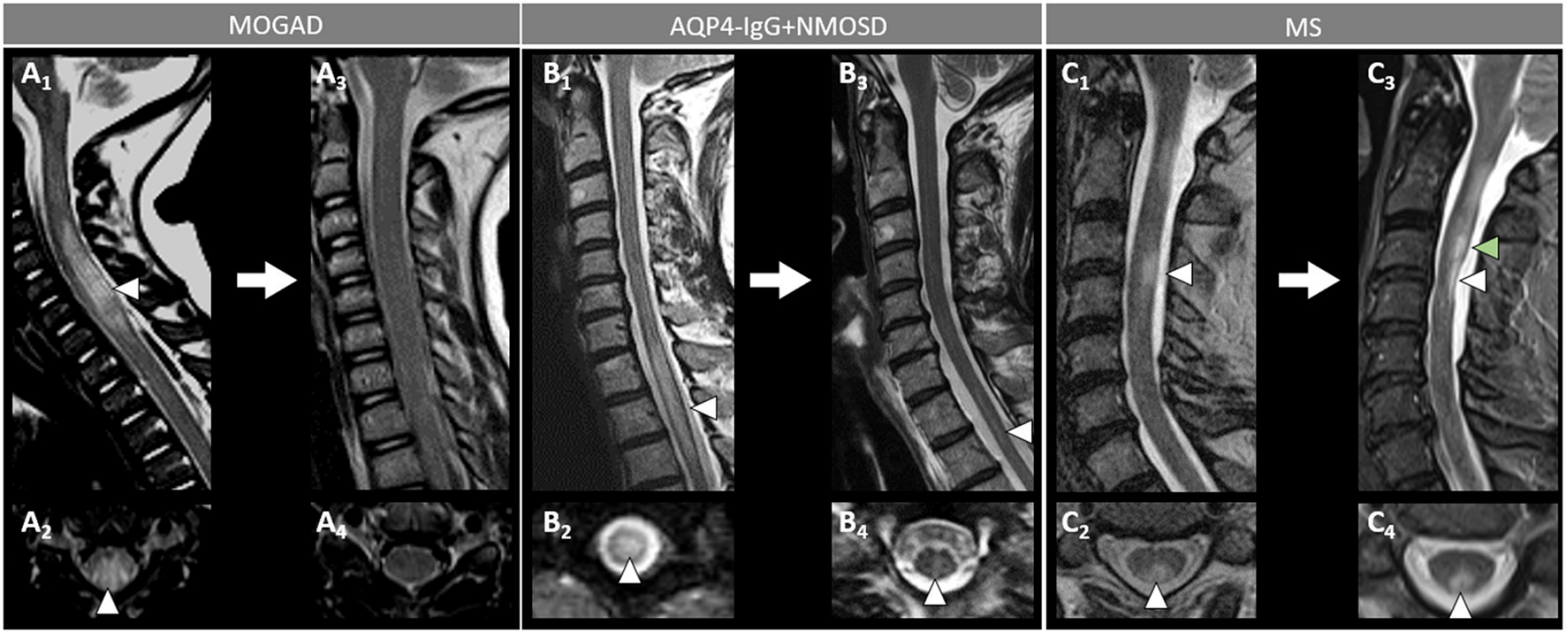
Spinal cord MRI lesion evolution in patients with MOGAD, AQP4-IgG+NMOSD, and MS on T2-images. [**(A)**: MOGAD) A longitudinally extensive T2-hyperintense spinal cord lesion with accompanying spinal cord swelling (A_1_, arrowhead) and gray matter involvement (A_2_, arrowhead), with complete resolution at follow-up (A_3_, sagittal, and A_4_, axial view). [**(B)**: AQP4-IgG+NMOSD] A longitudinally extensive T2-hyperintense (B_1_, arrowhead), centrally located thoracic spinal cord lesion (B_2_, arrowhead). The T2-lesion has substantially reduced in size at follow-up (B_3_, arrowhead), with only a mild residual hyperintensity still visible on sagittal view (B_3_, arrowhead) and axial view (B_4_, arrowhead). [**(C)**: MS] A short T2-hyperintense cervical cord lesion with accompanying spinal cord swelling (C_1_, arrowhead) and involving the posterior white matter tracts (C_2_, arrowhead), with residual T2-hyperintensity and local atrophy at follow-up (C_3_, sagittal view, white arrowhead, and C_4_, axial view, arrowhead). At follow-up, the patient also developed a new interval lesion (C_3_, green arrowhead). MOGAD, myelin oligodendrocyte glycoprotein antibody-associated disease; AQP4-IgG+NMOSD, aquaporin-4-IgG seropositive neuromyelitis optica spectrum disorder; MS, multiple sclerosis.

After the presenting attack, approximately 40%-50% of MOGAD patients maintain a monophasic course while 50–60% of cases experience disease relapses (22, 23, 63, 90). The persistence of MOG-IgG serum positivity after the first attack increases the likelihood of subsequent relapses, although relapses can also be observed in a minority of patients who become seronegative. In addition, many patients that are persistently positive can remain monophasic and therefore an exact prediction of the disease course at presentation is not possible based on MOG-IgG titers (23, 30, 91–94). An important characteristic of MOGAD MRI lesions is the tendency to resolve completely after the acute attacks, resulting in a complete normalization of the brain and/or spine MRI in 50–80% of cases (30, 55, 60). This represents a major difference from AQP4-IgG+NMOSD and MS where complete lesion resolution is extremely rare (Figures 5, 6). Similarly, the occurrence of new asymptomatic T2-lesions is rare in MOGAD (approximately 3% of cases), suggesting absence of disease activity between attacks (95, 96). This also suggests that serial MRI using conventional sequences in MOGAD is of limited utility for monitoring of disease activity in both clinical trials and clinical practice. This further differentiates the disease from MS where serial MRI have high yield of capturing silent disease activity between attacks, although is less common in the era of high efficacy MS disease modifying treatments. A few studies using nonconventional MRI sequences at sites of T2-lesion resolution show values of fractional anisotropy, mean diffusivity, and magnetization transfer ratio comparable to those of unaffected controls, suggesting absence of residual structural damage (97–99). However, reduction in gray matter volumes (cortex, deep gray nuclei, and spinal central gray matter) has been reported in patients with relapsing disease or persistent T2-abnormalities, highlighting the importance of preventing relapses in MOGAD (98, 99). Diffusion tensor imaging in non-lesional white matter in MOGAD do not show structural abnormalities (99).

Available data on the long-term clinical outcomes in MOGAD patients are in keeping with the above-mentioned MRI findings, showing complete or nearly complete recovery from attacks in most cases (82, 84, 100). However, residual cognitive deficits may occur even in children, and a minority of MOGAD patients have an unfavorable functional outcome (101–104). In one series of 29 patients followed for a median of 14 (range, 9–31) years, the median EDSS at last clinical follow-up was 2, with only two cases (7%) having an EDSS of ≥6 (100, 101). Another study on 61 patients followed for a median of 15 (range, 8–55) years, reported a median EDSS at last follow-up of 1 (range, 0–7.5) with 12.5% of cases having an EDSS of ≥6. In the same study, 16% of patients remained blind in at least one eye (105). In most patients with MOGAD ON, recovery of visual acuity is generally very good, although irreversible visual loss and blindness may occur in a minority of cases (100, 105, 106). Despite the severity of acute MOGAD attacks being similar to AQP4-IgG+NMOSD, the complete or nearly complete recovery observed in the majority of MOGAD cases is more similar to MS. In contrast to MS, however, a secondary progressive disease course is extremely rare in both AQP4-IgG+NMOSD and MOGAD and should prompt considering alternative etiologies (see also “*Atypical clinical-MRI phenotypes and risk of false positivity*” below) (100).

### Cerebrospinal Fluid Findings

Lumbar puncture during MOGAD attacks reveals CSF pleocytosis (>5 white blood cells/mm^3^) in >50% of patients, with important variability based on the specific attack phenotype (isolated optic neuritis, 16%; isolated myelitis, 74%; isolated brain/brainstem attacks, 72%; multifocal CNS involvement, 50–80%) (107–109). The pleocytosis is marked (>50 white blood-cells/mm^3^) in approximately 30% of patients acutely, a very uncommon finding in MS but that can be similarly seen in AQP4-IgG+NMOSD (108, 109). Also similar to AQP4-IgG+NMOSD, CSF-restricted oligoclonal bands (OCB) are rare and found in only approximately 15% of cases and may be transient. This is very different from MS where OCB are found in approximately 85% of cases and persist over time, except for some specific circumstances (110).

The cytokine profile in MOGAD during acute attacks is similar to that of AQP4-IgG+NMOSD, with predominant representation of Th17-related cytokines (e.g., IL-6, IL-8, IL-17) that may also serve as potential therapeutic targets (111). This differs from the Th1-related cytokines mainly observed in MS (112). CSF and serum levels of glial fibrillary acidic protein (GFAP), a marker of astrocytic damage, are generally lower in MOGAD compared to AQP4-IgG+NMOSD, which is in line with the different antibody cell target (oligodendrocyte vs. astrocyte) (113, 114). In all these three diseases, however, CSF levels of neurofilament light chains (a marker of neuroaxonal damage) increase during attacks, in particular at onset, and can also be detected in serum by using ultra-sensitive techniques, suggesting indirect neuronal damage in both conditions (115–118).

### Coexisting Autoimmunity

Other neural autoantibodies can rarely be detected in association with MOG-IgG. In these patients, the clinical-MRI phenotype seems predominantly driven by the accompanying autoantibody, although some features may overlap with MOGAD (119). Antibodies against the N-methyl-D-Aspartate receptor (NMDA-R-IgG) are the most commonly encountered in association with MOG-IgG, and patients typically present with encephalopathy, seizures, and leptomeningeal enhancement (119, 120). A double positivity for AQP4-IgG and MOG-IgG is extremely rare (0.06% of cases tested by live cell based assay [CBA]), with MOG-IgG generally at low titer, and patients typically following a AQP4-IgG+NMOSD phenotype (45).

Systemic non-organ and organ specific autoantibodies are generally not observed in patients with MOGAD, which is different from AQP4-IgG+NMOSD where the association with systemic autoantibodies is stronger (121, 122). Nonspecific positivity for autoantibodies that are relatively common in the general population (e.g., low titer glutamic acid decarboxylase (GAD)-65-IgG) can rarely be detected due to passive transfer after administration of hemoderivates (*e.g*., intravenous immune-globulins) (123).

### Optical Coherence Tomography

**Figure 7:**
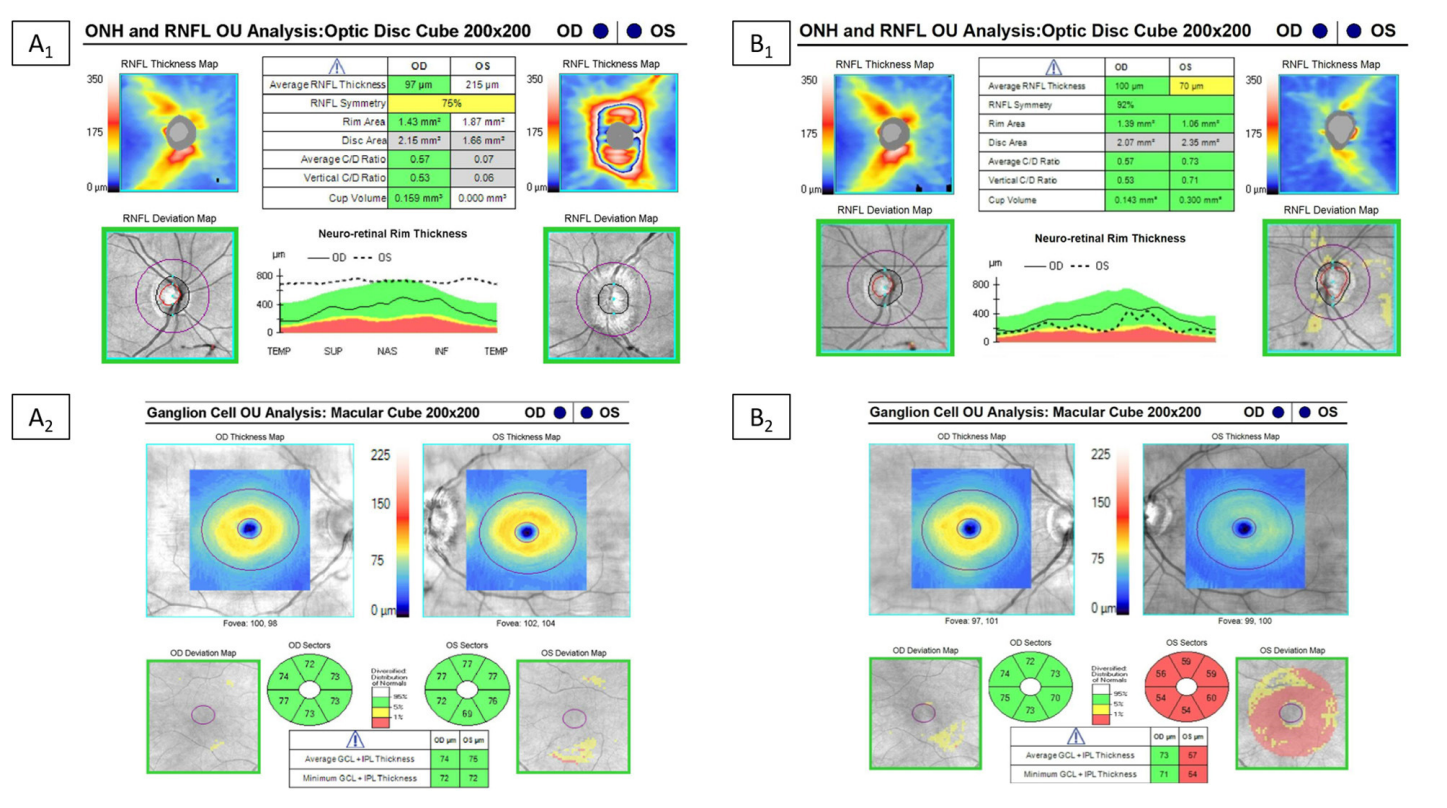
Example of optical coherence tomography alterations in patients with MOGAD ON. Optical coherence tomography in a MOGAD patient with optic neuritis in the left eye. Left images **(A1,A2)** are the OCT images at the time of the acute optic neuritis, which shows significant peripapillary retinal nerve fiber layer thickening (RNFL) in the left eye and a normal ganglion cell-inner plexiform layer (GC-IPL) thickness. Images on the right **(B1,B2)** are the repeat OCT 1 year after the optic neuritis attack, which shows thinning of the peripapillary RNFL and macular GC-IPL in the left eye despite recovery back to a visual acuity of 20/20.

Optical coherence tomography (OCT) is an essential part of the diagnostic evaluation of optic neuritis and this imaging diagnostic test can be useful for confirming an optic neuropathy as well as potentially discriminating between MOGAD, AQP4-IgG+NMOSD and MS (124–128). In the acute setting of MOGAD optic neuritis, the peripapillary retinal nerve fiber layer (pRNFL) is often significantly thickened, and indeed the median thickness was greater in MOGAD at 164 μm vs. MS at 103 μm in one study (129). Over 3–6 months after optic neuritis, there is progressive thinning of the pRNFL and the macular ganglion cell and inner plexiform layer (mGCIPL); the thinning of the mGCIPL tends to occur earlier and within a few weeks of the attack while the pRNFL thinning takes longer to develop possibly due to the slowly resolving optic nerve head edema (130). Severe thinning of these layers often occurs in MOGAD optic neuritis from recurrent attacks, while AQP4-IgG+NMOSD tends to cause significant thinning after single attacks (Figure 7). One of the initial large studies on OCT in optic neuritis demonstrate that thinning of the pRNFL below 75 μm was associated with worse visual outcomes (131), however some patients can have significant pRNFL and mGCIPL thinning and retain good visual function (132), especially in MOGAD patients (133–135). While optic neuritis from AQP4-IgG+NMOSD is known to cause more severe vision loss, some studies have shown similar amounts of thinning of the pRNFL and mGCIPL despite this discordant visual outcome (125, 135). The cause of the discrepancy between the detected structural abnormalities and different functional impairment in the two diseases remains unclear, and has been speculated to be related to the pathophysiology of AQP4-IgG+NMOSD being an astrocytopathy with potential for more severe retinal dysfunction (135). Another possibility is that the pRNFL and mGCIPL becomes “bottomed out” at around 50-60 μm and therefore OCT may not capture the greater extent of optic nerve damage that may occur in AQP4-IgG+NMOSD optic neuritis compare to MOGAD.

## Diagnosis

A correct diagnosis of MOGAD requires: (1) detection of MOG-IgG in serum and/or CSF with a reliable laboratory assay; and (2) presence of a clinical-MRI phenotype compatible with MOGAD (23, 136). Positivity for MOG-IgG in patients with atypical phenotypes should raise the suspicion for a false positive result, which may have important treatment implications (137). As MOG-IgG titer can decrease to undetectable in 30–40% of cases (and MRI abnormalities resolve completely) after acute attacks (23, 30, 55), the diagnosis of MOGAD is sometimes not possible outside of the acute setting. In similar cases, testing MOG-IgG on stored serum and/or CSF samples obtained at the time of the attack (when available) is useful.

### MOG-IgG Testing

Demonstration of MOG-IgG positivity with a reliable assay is crucial for a correct MOGAD diagnosis and to reduce the risk of false positive results. Live cell-based assays (CBA) using full-length human MOG are optimal, and typically include: (1) a fluorescence activated cell sorting (FACS) assay providing a quantitative fluorescence ratio between MOG transfected vs non-transfected cells; or (2) a visual assessment of transfected cells using a fluorescence microscope (138, 139). While these assays have consistently shown a very high (≈99%) specificity for typical MOGAD phenotypes in multicentre comparative studies, a correct assessment of their sensitivity is limited by the lack of a reference diagnostic gold-standard for comparison. A slightly lower specificity (≈98%) is reported for the commercially available cell-based assay using fixed transfected cells (Euroimmun), where the natural spatial conformation of the MOG protein can be altered by the fixation process hampering its recognition by MOG-IgG (138, 139). The sensitivity of fixed CBA is also lower when compared to the live CBA. However, a fixed CBA is still significantly superior to other non-CBAs, such as ELISA, which cannot reliably identify MOG-IgG.

Testing for MOG-IgG is generally recommended in serum, especially initially during the diagnostic work-up in patients suspected of having MOGAD. Concomitant serum and CSF positivity is not uncommon. Isolated CSF positivity for MOG-IgG is a rare but recognized possibility (37, 140–143). Therefore, CSF testing should be considered in patients with clinical-MRI features suggestive of MOGAD but negative results on serum testing. However, similar to the occurrence of serum MOG-IgG false positives, rare CSF false positive results have been encountered in other diseases such as MS and thus a positive result always needs to be interpreted within the context of the clinical and MRI phenotype (see also “*Atypical clinical-MRI phenotypes and risk of false positivity*” below).

### Atypical Clinical-MRI Phenotypes and Risk of False Positivity

Importantly, antibody positivity is not always sufficient to guarantee a correct diagnosis of MOGAD (144). Despite the high specificity of CBAs for MOG-IgG, false positive results may occur, especially when the test is performed indiscriminately in large unselected populations (137, 145). When MOG-IgG testing is ordered, a thorough phenotypical assessment is mandatory to determine the pre-test probability. Patients with clinical-MRI phenotypes typically recognized to be associated with MOGAD (see also “*Clinical-MRI attributes*” above) are considered to have a high pre-test probability and a low risk of false positive results (137). On the other hand, patients presenting with clinical-MRI features that are generally not seen in MOGAD or that are more suggestive of an alternative diagnosis (low pre-test probability) have a significantly higher risk of false positive results. One study on 1,260 patients consecutively tested for MOG-IgG over 2 years at Mayo Clinic found a rate of false positive results of 28% (26/92 positive results) by using FACS CBA, corresponding to a positive predictive value of 72% despite a specificity of 98% (137). The positive predictive value was significantly higher after stratification for pre-test probability (high, 85%; low, 12%) and antibody titer (≥1:1,000, 100%; 1:100, 82%; 1:20 or 1:40, 51%), highlighting the importance of a correct phenotypical assessment before testing. In another study assessing MOG-IgG in all neurology patients admitted to hospital regardless of diagnosis, MOG-IgG was detected in approximately 1% of patients with other neurologic diseases and generally at low titer (145).

Interestingly, false MOG-IgG positivity may occur in patients with various alternative neurologic disorders (e.g., neoplastic, genetic, metabolic, vascular) but seems rare in subjects without known neurologic diseases. This might suggest MOG-IgG production may increase in the context of epitope spreading from neurologic damage due to other etiologies, or cross-reactivity with autoantibodies directed against alternative targets. In patients with false MOG-IgG positivity, MS is generally the most common alternative diagnosis. This is due to the higher frequency of MS among demyelinating CNS disorders compared to MOGAD (approximately 40-50 times higher), and the common tendency among physicians to routinely test MS patients for MOG-IgG in order to exclude other diagnostic possibilities. Although patients with MS phenotype and MOG-IgG positivity are sometimes regarded as having “atypical manifestations of MOGAD that can mimic MS,” their clinical characteristics (e.g., presence of CSF-restricted OCB, persistence of T2-abnormalities over time, development of progressive disability) and response to MS-targeted disease modifying treatments are more suggestive of a false positive (or true positive but clinically irrelevant) result (146). This is also in line with rare cases of MOG-IgG coexisting with other autoantibodies (e.g., AQP4-IgG, NMDAR-IgG) where the clinical-MRI phenotype is typically predicted by the accompanying antibody, although overlapping features may occur (see also “*Coexisting autoimmunity*” above) (45, 147). In conclusion, routine MOG-IgG testing should strongly be avoided in patients with clinical-MRI features typical of MS. Other major red flags that should prompt considering MOG-IgG positivity as a false positive are listed in Table 2.

**Table 2:** Major red flags for false MOG-IgG positivity in patients with suspected CNS demyelinating syndromes.

Clinical features and disease course	- Progressive disease course, either from onset (primary progressive course) or after an initial period of relapsing disease activity (secondary progressive course). Consider MS, spinal cord sarcoidosis, metabolic, or genetic etiologies (e.g., B12 deficiency, adrenoleukodystrophy). - Hyperacute severe presentation (<12 h). Consider stroke/ischemic damage (e.g., spinal cord infarction, ischemic optic neuropathy). - Concomitant peripheral neuropathy. Can rarely be found in patients with MOGAD but its significance is unclear. Consider other causes of myeloneuropathy or encephalomyeloneuropathy (e.g., other autoimmune/paraneoplastic neurologic disorders, metabolic or genetic conditions).
CSF findings	- Oligoclonal bands or elevated IgG-Index. Consider MS or sarcoidosis.
MRI	- Brain abnormalities typical of MS: Ovoid periventricular lesions in the hemispheric white matter (perpendicularly oriented to the main ventricle axis), brainstem, or cerebellar hemispheres; linear or S-shaped juxtacortical lesions. Ring or open-ring enhancement. - Spinal cord abnormalities typical of MS: Multiple, short (<3 contiguous vertebral body segments) lesions peripherally located on axial images (commonly along the dorsal-lateral columns), often accompanied by focal atrophy. Ring or open-ring enhancement. - Lesion persistence, or development of new asymptomatic brain/spine lesions over time. Consider MS. - Central vein sign. Consider MS.
Serology	- Borderline or low MOG-IgG titer. These should be interpreted based on the clinical-MRI phenotype. Consider repeat testing, preferably with a more accurate assay (e.g., live vs. fixed CBA). Consider CSF testing when the diagnostic suspicion is high.^*^ - Coexistence of neural autoantibodies other than MOG-IgG. In these patients the contribution of MOG-IgG to the neurological syndrome is generally poor and the clinical-MRI phenotype is mostly driven by the accompanying antibody.
Other	- Other clinical-MRI findings suggestive of alternative diagnosis (e.g., blood or microhemorrhages on MRI, positive screening for infections on serum and/or CSF).

*CBA, cell-based assays; CSF, cerebrospinal fluid; MOGAD, myelin oligodendrocyte glycoprotein-IgG associated disease; MS, multiple sclerosis*.

* *The validity of CSF-exclusive MOG-IgG positivity in conferring a diagnosis of MOGAD is still pending validation*.

### Special Settings Potentially at Higher Risk of Neurological Autoimmunity

A number of emerging settings putting patients potentially at higher risk for development of neurological autoimmunity have recently been reported, and some are applicable to MOGAD:

1. Neurological immune-mediated disorders of any type (including CNS demyelination) are a well-established complication of treatment with immune-checkpoint inhibitors (148, 149). Despite a number of different neural antibodies having been described in patients treated with these drugs, the association with MOG-IgG seems particularly scarce and deserves further dedicated investigations.
2. Cases of MOGAD have recently been reported following both SARS-CoV-2 infection and vaccination (150, 151). Although prodromal episodes of infection or vaccination are known to occur in up to one third of MOGAD patients (53), the overall risk of developing MOGAD or a MOGAD relapse after SARS-CoV-2 infection/vaccination appears to be extremely low (152), given the extremely high prevalence of the infection and vaccination without an associated observed large increase in MOGAD cases (153). Thus, most experts believe the benefits of COVID-19 vaccination far outweigh this extremely rare possibility.
3. Treatment with TNF-inhibitors has been associated with an increased risk of CNS demyelination (154). Although this association seems particularly true for MS and antibody-negative CNS demyelinating disorders, onset of MOGAD in patients exposed to TNF-inhibitors seems rare (155).
4. Neurological autoimmunity has been reported in post-transplant patients and patients treated with alemtuzumab (156, 157). In both cases, imbalance between B- and T-cell reconstitution after aggressive immunosuppression may rarely result in autoimmunity, including MOGAD (156).
5. Little is known about the risk of MOGAD relapses during pregnancy and post-partum. Small case series suggest a reduction in relapse rate during pregnancy compared to the pre-pregnancy period similar to other autoimmune conditions. The frequency of relapses increases again in the post-partum period and is lower in those receiving immunosuppressive treatment (158, 159).
6. In contrast to other neural antibodies, MOG-IgG does not have a strong paraneoplastic association and therefore MOGAD patients should not be routinely screened for cancer. However, cases of paraneoplastic MOGAD have seldomly been reported with evidence of MOG expression in the neoplastic tissue (160).

## Management

There are no randomized controlled trials available in MOGAD and existing recommendations for treatment are mostly empirical, derived from existing data on AQP4-IgG+NMOSD, and/or based on retrospective studies. Interestingly, some drugs that are typically very effective in AQP4-IgG+NMOSD, such as rituximab, seem less effective in MOGAD, further highlighting the need for dedicated randomized controlled trials.

### Treatment of Disease Attacks

Similar to other demyelinating and non-demyelinating inflammatory disorders, disease flares in MOGAD are generally treated with high dose corticosteroids and most patients respond briskly to this. A retrospective study including both AQP4-IgG+NMOSD and MOGAD suggested that earlier treatment may lead to better outcomes (161). In patients with severe attacks and high disability at attack nadir, early initiation of a combination of intravenous corticosteroids and PLEX is reasonable. The efficacy of PLEX in patients with severe demyelinating attacks who fail to recover after intravenous corticosteroids was first proven in 1999 with a seminal randomized, sham-controlled, double-masked trial (162). Data on the use of IVIg for MOGAD attacks are limited so that IVIg may represent a reasonable treatment option after PLEX in very severe/refractory cases. Testing for MOG-IgG and other antibodies on serum is preferred before treatment initiation as acute immunotherapy may reduce MOG-IgG titer to undetectable (23, 30). After samples of serum have been obtained, acute treatment should promptly be initiated while waiting for antibody test results.

Commonly used dosages and types of acute treatment include:

- Corticosteroids - Intravenous methylprednisolone (IVMP) 1,000 mg once daily for 5 days is the standard treatment for a MOGAD attack. Oral prednisone 1250 mg (25 × 50 mg tablets) once daily for 5 days is an alternative and equivalent to the 1,000 mg IVMP dose but swallowing 25 tablets daily can sometimes pose a challenge for some patients. Common side effects include hyperglycaemia (more relevant in diabetic patients) and steroid-induced psychosis. A slow taper with oral prednisone over 6–8 weeks is sometimes considered to prevent early relapse but needs to be balanced by the risk of side effects and further studies are needed to evaluate this approach (25, 90).
- IVIg – Typically 0.4 g/Kg/day for 5 days. In some studies, IVIg have been associated with hyperviscosity and an increased risk of thromboembolic events, especially in patients at risk (163). Renal failure and aseptic meningitis may also rarely occur, but are generally preventable with sufficient hydration and slow infusion rate (163). Serum IgA levels should be obtained before the first administration to prevent IgA deficiency-related anaphylactic reactions (163).
- PLEX – Typically 5–7 exchanges every other day. PLEX is generally well-tolerated and safe, although often requires a central line and rare cases of severe vascular hypotension and cardiac arrythmias have been reported (164). PLEX-associated infections have also been reported rarely and may be severe (potentially related to both the central line placed and the treatment-associated immunosuppressive effect) (165).

In rare cases refractory to the conventional acute treatments, a more aggressive immunosuppression with cyclophosphamide, rituximab or tocilizumab can be considered.

### Maintenance Treatment

Given the high frequency of patients with a monophasic course (40–50%), and the generally good recovery from disease attacks, the decision of initiating a long-term immunosuppressive treatment in MOGAD should carefully be discussed on a patient-by-patient basis. In general, maintenance attack-prevention immunotherapy is offered to those that have had two or more attacks, but not initiated after the first attack (including in those persistently positive for MOG-IgG) to avoid over treatment of monophasic disease. However, exceptions are considered in cases of severe residual deficits following the presenting attack, to prevent further disability (e.g., preserve vision in patients with residual monocular blindness after the initial attack).

Common long-term treatment options include:

- Maintenance IVIg – Maintenance infusions of IVIg (loading dose of 0.4 g/Kg/day for 5 consecutive days, followed by treatment every 4 weeks with a dose of 0.4 g/kg to 2 g/kg) can be considered, especially in pediatric patients or in patients with higher risk of infections, in whom avoiding long-term immunosuppression might be preferrable. In one retrospective series of 70 MOGAD patients, the relapse rate in those receiving periodic IVIg infusions (20%) was significantly lower compared to patients receiving azathioprine (59%), rituximab (61%), and mycophenolate mofetil (74%) (166). Another retrospective study on 59 adult patients with MOGAD (58 with relapsing disease) found a significant reduction in annualized relapse rate during treatment with IVIg compared to the pre-IVIg period. The results were similar in patients receiving IVIg as first-line treatment and those who initiated IVIg after failure of other immunotherapies. The risk of relapse was lower in those treated with higher IVIG doses or more frequently (e.g., 0.4 g/kg every week, or 2 g/kg every 4 weeks). Two patients (3%) experienced worsening disability (EDSS score) during IVIG treatment (167). The high costs and restricted availability are the main elements that limit the use of this treatment in a larger scale. Subcutaneous Ig has recently been reported to be safe and effective in preventing relapses in a series of six MOGAD patients, and may represent an advantageous treatment option due to the better tolerability; subcutaneous immune globulin is self-administered and IVIg can also often be given at home when home infusion services are available (168).
- Rituximab (anti-CD20 monoclonal antibody) – Despite its high utility in preventing relapses in AQP4-IgG+NMOSD, the efficacy of rituximab in patients with MOGAD seems less (169), with up to one third of patients or more expected to experience relapses despite full B-cell depletion (170). Nonetheless, rituximab remains a potential treatment option for MOGAD, with higher efficacy reported when the drug is administered as first-line maintenance therapy (166, 170). In adults, rituximab is typically administered in one of the following two regimens: 1) 375 mg/m^2^ of body surface area weekly for four consecutive weeks (induction), followed by 375 mg/m^2^ weekly for 2–4 weeks at the time of reinfusion; or 2) 1 g/week repeated after 2 weeks (induction), followed by reinfusions (generally 1 g x 2 doses separated by 2 weeks, single 1 g reinfusion, or 375 mg/m^2^/week for two consecutive weeks). Since the B-cells usually start to repopulate 8–12 months after rituximab administration, the timing of reinfusions can either be predetermined (generally at fixed 6 months intervals), or guided by periodic monitoring of blood CD19+ B-cells (every 6–8 weeks) until their value re-increases over 1% of total mononuclear cells (171). Since some cases with NMOSD experience relapses despite CD19+ cell-depletion, monitoring of CD27+ memory B-cells has been proposed (although not commercially available) (172). Rare cases of prolonged B-cell depletion after rituximab have been reported (173). Rituximab is generally well tolerated and safe. The most common adverse events include infusion-related reactions and increased risk of infections long-term (with a higher risk in patients with prior history of immunosuppression, lymphopenia or hypogammaglobulinemia) (174, 175). Rituximab treatment seems to increase the risk of severe COVID-19 infections and reduce the effect of COVID-19 vaccines, although an increased mortality was only observed in patients with NMOSD and comorbid conditions (176–178).
- Azathioprine (generally administered at the dose of 2–3 mg/Kg/day) and mycophenolate mofetil (generally administered at the dose of 600 mg/m^2^ or 2 g daily in divided doses) can also be considered for long-term immunosuppression in MOGAD (166, 179, 180). Advantages of these medications include the oral route of administration, their availability and lower costs compared to the other treatment options. A disadvantage of these drugs is the long time (usually several months) from treatment initiation to action. During this time, oral prednisone can be continued and later tapered, but this can increase the side effect burden. These medications are associated with an increased risk of infection and long-term use can be associated with an increased risk of hematologic and skin malignancies.
- IL-6 targeting treatments (e.g., tocilizumab, satralizumab) – Small case series suggest tocilizumab might be highly effective in patients with MOGAD refractory to other immunosuppressive treatments (181, 182). The drug is generally administered at a dose of 8 mg/Kg monthly, for a maximum recommended dose of 800 mg/month in adults. A multicenter randomized controlled trial is currently underway to evaluate the efficacy of satralizumab in MOGAD.
- Future treatment directions: (1) B-cell depleting monoclonal antibodies other than rituximab have poorly been studied in MOGAD. These include other anti-CD20 agents (e.g., ocrelizumab, ofatumumab) and anti-CD19 agents (e.g., inebilizumab) (183, 184). These drugs can reasonably be considered alternatives to rituximab in MOGAD due to the similar mechanisms of action. In AQP4-IgG+NMOSD, inebilizumab was shown to be effective in reducing relapses in a randomized clinical trial (185). (2) Eculizumab (anti-C5) has been proven very effective for relapse prevention in AQP4-IgG+NMOSD in a recent randomized trial (186), but very little data exist on its potential utility in MOGAD, and complement has not been definitively proven to be integral to the pathogenesis of MOGAD. This drug might be considered as a second-line agent for very refractory cases (33, 39), but there are no published case series reporting its treatment in MOGAD to date. (3) Rozanolixizumab (anti-neonatal Fc receptor) – Blocking of the neonatal Fc receptors favors degradation of pathogenic autoantibodies. This mechanism has been proven to be effective in patients with myasthenia gravis and antibodies against the acetylcholine receptor (187), and a randomized placebo controlled clinical trial is underway in MOGAD.

### Quality of Life and Supportive Treatment

Chronic pain and depression have been reported in up to 51 and 42% of patients with MOGAD, respectively, and have a significant impact on quality of life (188). Pain can be neuropathic, spasticity-associated, and/or secondary to painful tonic spasms (i.e., episodes of intense pain that accompany tonic postures of one or more limbs lasting 30–60 s). Given the strong correlation between pain and depression in these patients, effective treatment of pain can indirectly have a beneficial effect on depression, and vice versa (188, 189). In the absence of dedicated trials, common pain medications include non-opioid analgesics, antidepressants (e.g., duloxetine), and antiepileptic agents (e.g., gabapentin, pregabalin). Painful tonic spasms in particular generally respond well to low dose carbamazepine (200–300 mg/day) although are more common with AQP4-IgG+NMOSD than MOGAD (190). Initiation of immunosuppressive treatment has also been reported to improve pain (188, 189). Muscle relaxants (e.g., baclofen, benzodiazepines) and physical rehabilitation should be offered for spasticity. A more detailed description of the different treatment options in MOGAD is beyond the scope of this review article but has been summarized elsewhere (191).

## Conclusions

MOGAD is now recognized to be a distinct demyelinating CNS disorder, different from MS and AQP4-IgG+NMOSD. Awareness of the clinical-MRI characteristics of MOGAD is fundamental for prompt diagnosis and treatment. The disease is defined by MOG-IgG which is a highly specific biomarker, but caution is needed with the interpretation of low titers and atypical phenotypes, as false positives can occur. Although the prognosis is generally favorable, severe residual disability can occur in MOGAD, highlighting the importance of attack prevention in patients with relapsing disease. Major unmet needs for future studies include early identification of patients at higher risk of relapsing disease and/or permanent disability, and identification of effective acute and long-term treatments through dedicated randomized clinical trials.

## Author Contributions

ES and EF study concept, design, and drafted the manuscript and figures. LC and JC drafted the figures and revised the manuscript for intellectual content. SM, GF, AD, AL-C, and SP revised the manuscript for intellectual content. All authors contributed to the article and approved the submitted version.

## Funding

EF has received funding from the NIH (R01NS113828).

## Conflict of Interest

The authors declare that the research was conducted in the absence of any commercial or financial relationships that could be construed as a potential conflict of interest.

## Publisher's Note

All claims expressed in this article are solely those of the authors and do not necessarily represent those of their affiliated organizations, or those of the publisher, the editors and the reviewers. Any product that may be evaluated in this article, or claim that may be made by its manufacturer, is not guaranteed or endorsed by the publisher.
